# SERa-Positive Oocyte Intracytoplasmic Injection and Its Outcome: A Case Report

**DOI:** 10.7759/cureus.58836

**Published:** 2024-04-23

**Authors:** Neeraj Vishwakarma, Charu Pareek, Nancy Nair, Ankit Badge, Pranita A Bawaskar, Avanti Kalbande

**Affiliations:** 1 Clinical Embryology, Datta Meghe Institute of Higher Education & Research (Deemed to be University), Wardha, IND; 2 Microbiology, Datta Meghe Institute of Higher Education & Research (Deemed to be University), Wardha, IND; 3 Obstetrics and Gynaecology, Datta Meghe Institute of Higher Education & Research (Deemed to be University), Wardha, IND; 4 Obstetrics and Gynaecology, Shalinitai Meghe Hospital & Research Centre, Nagpur, IND

**Keywords:** in vitro fertilization, embryo transfer, smooth endoplasmic reticulum aggregates, granulocyte macrophage colony-stimulating factor, intracytoplasmic sperm injection

## Abstract

This case report describes the use of smooth endoplasmic reticulum aggregates-positive (SERa+) oocytes along with intracytoplasmic sperm injection (ICSI), supplemented with granulocyte-macrophage colony-stimulating factor (GM-CSF), aiming to enhance fertilization rates and reproductive outcomes. A 39-year-old woman, facing primary infertility for the past seven years, received assisted reproductive treatment (ART), which included adding GM-CSF to the culture medium and culture SERa+ oocytes before ICSI. Clinical results, embryo quality, fertilization rates, and other fertility parameters were used to track the patient's progress toward this individualized approach that led to a positive twin pregnancy and healthy twin babies.

## Introduction

Infertility is defined as the inability of a couple to conceive after more than a year of unprotected intercourse. In females, disorders of the endocrine system, fallopian tubes, uterus, ovaries, and other organs may result in infertility [1]. Infertility is a medical condition that affects 10-15% of couples, sending them through a range of emotions, including frustration, desperation, and anxiety [2]. Psychological conditions such as depression, stress, eating disorders, sleep difficulties, and addictions may play a role in idiopathic infertility [3].

Idiopathic infertility occurs when the exact cause of infertility cannot be determined. The appearance of smooth endoplasmic reticulum aggregates (SERa) can be considered one of the idiopathic reasons for infertility since there is no recognition of the mechanism of SERa formation, and information on the likelihood of SERa oocyte recurrence is inconsistent and not sufficiently clear to establish a distinct constitutive origin [4].

Morphological evaluation of oocytes intended for assisted reproduction is one of the main predictive factors for successful fertilization, implantation, and pregnancy [5]. After denudating the oocyte for intracytoplasmic injection (ICSI), embryologists often find many oocyte dysmorphisms. Special consideration is given to oocytes displaying cytoplasmic characteristics, including changed cytoplasmic granularity, vacuole formation, lipofuscin structures, and visible SERa [6].

The endoplasmic reticulum (ER) is an organelle that comprises an interconnected network of flattened tubes or membrane-enclosed sacs. Furthermore, during the early stages of cleavage, the ER and mitochondria complex is necessary for synthesizing lipids and triglycerides, accumulating energy, and creating nuclear and cytosolic membranes [5]. The presence of abnormally large aggregates of ER in oocytes is associated with a low quality of embryos, a fertilization rate, and a low implantation rate [7]. It is possible for embryos created from SERa+ oocytes to develop into normal healthy newborns, and there is no absolute negative correlation between SERa+ oocyte, cycle, embryological, clinical, and neonatal outcomes [8,9].

The number of oocytes that remain in the ovary can be predicted using hormones such as follicle-stimulating hormone (FSH), estradiol (E2), anti-Mullerian hormone (AMH), and early follicular phase inhibitor B [9]. AMH is a dimeric glycoprotein that is a member of the transforming growth factor beta family and is produced by granulosa cells of pre-antral, secondary, and early antral follicles (≤6 mm). AMH levels are inversely correlated with age due to the primordial follicle pool eventually contracting as women approach menopause. The amount of gonadotropin administered, the duration of the stimulation of ovaries, and the serum estradiol concentration on the same day of ovulation trigger are positively connected with the presence of SERa+ in MII oocytes [10].

Granulocyte-macrophage colony-stimulating factor (GM-CSF) is a pleiotropic cytokine that belongs to the hematopoietic growth factor. It is a factor that contributes significantly to the success of pregnancies because it influences not only ovarian function and embryo implantation but also endometrial thickening and the pathophysiology of endometriosis, all of which are fundamentally linked to a decrease in pregnancy loss. GM-CSF influences and promotes oocyte maturation, endometrial receptivity, preimplantation embryo development, and trophoblast invasion. It appears to increase the likelihood of pregnancy among ART patients [11]. This case study reports one such case of successful implantation and live birth of twin babies from SERa+ oocytes.

## Case presentation

We received a case of an infertile couple seeking infertility treatment at our test tube baby center who has been suffering from primary infertility for the past seven years. The 41-year-old male and the female aged 39 years presented an advanced age that could be a predictive factor of infertility, but a complete diagnostic evaluation was carried out to deduce the actual reason. After enrolling at our center, both received a detailed explanation of the processes, benefits, and demerits, and their informed consent was obtained.

Medical history of the couple

A 39-year-old female and her 41-year-old spouse presented to our medical facility, having experienced infertility for seven years. The male partner has a 14-year history of hypertension and previously underwent two unsuccessful in vitro fertilization (IVF) cycles before seeking consultation. Their reproductive attempt has not resulted in pregnancy thus far despite regular unprotected sexual activity throughout this period. This situation has caused significant emotional strain on both individuals due to their inability to develop and implement effective therapeutic strategies and treatments.

Clinical findings

The male partner was advised to undergo a semen analysis. In the lab, a semen sample was collected. According to WHO 2021 guidelines, the results of the semen analysis reported normal parameters. The total sperm count was 21 million per ejaculate, and progressive motility was 35%, as shown in Table 1.

**Table 1:** Semen analysis report mL: millilitre; pH: potential of hydrogen; M: million; PR: progressive motility; NP: non-progressive motility

Semen parameter	Reference value	Findings
Volume	≥1.5 mL	1.2 mL
pH	7.2 to 8.0	7.8
Total sperm count	≥39 M/mL	21 M/mL
Motility (PR+NP)	≥40%	35%
Morphology	≥4%	14%

The laboratory results for the parameters of the female patient are as follows: LH levels were within the normal range at 3.2 mIU/mL (reference values: 2-10 mIU/mL), testosterone levels were 38 ng/dL (reference values: 15-70 ng/dL), progesterone levels were 0.33 ng/mL (reference values: 0.1-0.7 ng/mL), estradiol levels were 36 pg/mL (reference values: 30-400 pg/mL), FSH levels were 17.3 mIU/mL (reference values: 4.7-21.5 mIU/mL), prolactin levels were 18 ng/mL (reference values: <25 ng/mL), and AMH levels were 1.1 ng/mL (reference values: 1.5-4.0 ng/mL). These results provide valuable insights into the patient's hormonal profile, aiding in the assessment and management of her reproductive health, as indicated in Table 2.

**Table 2:** Hormonal profile of the female patient. LH: luteinizing hormone; FSH: follicle-stimulating hormone; AMH: anti-müllerian hormone; mIU: milli international units; ng: nanogram; dL: deciliter; pg: picogram

Parameters	Reference values	Results
LH	2-10 mIU/mL	3.2 mIU/mL
Testosterone	15-70 ng/dL	38 ng/dL
Progesterone	0.1-0.7 ng/mL	0.33 ng/mL
Estradiol	30-400 pg/mL	36 pg/mL
FSH	4.7-21.5 mIU/mL	17.3 mIU/mL
Prolactin	<25 ng/mL	18 ng/mL
AMH	1.5-4.0 ng/mL	1.1 ng/mL

Therapeutic interventions

The patients signed an informed consent prior to the start of the stimulation protocol. The woman underwent a gonadotropin-releasing hormone (GnRH) antagonist protocol. She received 187.5IU/day of human recombinant follicle stimulating hormone (rFSH) for the first five days; then 225 IU of human menopausal gonadotropin (hMG) and 0.25 mg of cetrorelix, administered subcutaneously for the next three days; and then finally on the 10th day, she received 0.25 mg cetrorelix, 0.2 mg of decapeptyl, and 5000 IU recombinant human chorionic gonadotropin (rHCG).

Oocyte pick-up was performed under transvaginal ultrasound guidance 36.5 hours after the dual trigger was administered, and a total of nine oocytes were retrieved, of which seven were metaphase II (MII) and two were metaphase I (MI). On proper microscopic assessment of these oocytes, aggregates of smooth endoplasmic reticulum were seen. Given this unique circumstance, the couple was informed about the possibility of performing ICSI with all seven SERa+ oocytes, which were retrieved. The couple denied donor oocyte. After understanding the potential outcomes, the couple decided to go with the ICSI procedure using the SERa+ oocytes. The oocytes were cultured in a fertilization medium after oocyte denudation until the next day. All of these seven oocytes were injected with spermatozoa obtained from fresh ejaculation by a male partner. The fertilized oocytes were cultured sequentially to enhance the blastocyst grade. After 16-18 hours of ICSI, the pronuclei were checked to confirm the fertilization outcome, and the fertilized oocytes (2PN) were placed in a cleavage medium until day 3 of the cleavage stage and subsequently in a blastogen medium with recombinant human cytokines GM-CSF until day 5 to enhance the blastocyst outcome (Table 3).

**Table 3:** Embryological assessment MI: metaphase I; MII: metaphase II; mm: millimeter

Characteristics	Value
Endometrial thickness	7.9 mm
Antral follicles count right and left	12
Number of oocytes retrieved	09
Number of immature oocytes - MI	02
Number of mature oocytes - MII	07
Number of fertilized oocytes	05
Number of unfertilized oocytes	02
Cleavage stage arrest	03
Blastocyst stage	02

A benchtop incubator with consistent humidity and gas content, i.e., pre-mixed gas CO_2_ of 6% and 5% O_2_, was used to culture the embryos. The embryos were simply cultured in a 30 µl culture medium. A single drop of culture medium of 30 µl for each oocyte was made. The quality of the oocytes was evaluated, and the embryos were observed on day 1, day 3, and day 5 stages. By the time of day 3, there were six to eight aggregated cells in each embryo. Day 5 blastocysts were graded according to the Gardner grading system, and the results are shown in Table 4.

**Table 4:** Day-wise embryo development 3: size of the expansion; A: trophectoderm; B: inner cellular mass; PN: pronuclei

Day 0	Day 1	Day 2	Day 3	Day 4	Day 5
07	5x 2PN	5x4 cell	4x8 cell	2x compaction	2x early blastocyst (3AB,3BB)

Morphological Assessment and Embryo Culture

**Figure 1:**
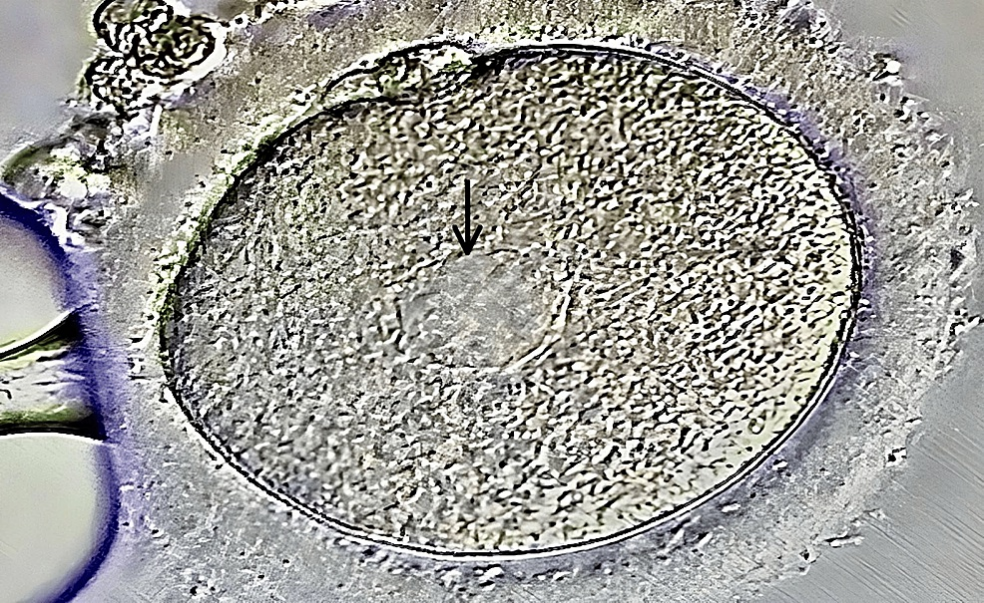
Smooth endoplasmic reticulum in an oocyte Black arrow: smooth endoplasmic reticulum aggregations

On the day of oocyte recovery, the presence of SERa within the ooplasm of all MII oocytes was recorded using an inverted contrast microscope. The large SERa in the cytoplasm of MII resemble vacuoles, but they can be easily distinguished because they are not filled with fluid and are not membrane-separated from the rest of the cytoplasm, as shown in Figure 1.

Cleavage rate is defined as the total number of day 3 embryos by the total number of fertilized oocytes multiplied by a hundred. Table 5 shows the fertilization rate of 71%, and the cleavage rate is just 57%, whereas the normal cleavage rate for ICSI is around 63%. On day 5, two embryos were frozen.

**Table 5:** Progress of embryo development

Development rate	Percentage
Fertilization rate	71%
Cleavage rate	57%

**Figure 2:**
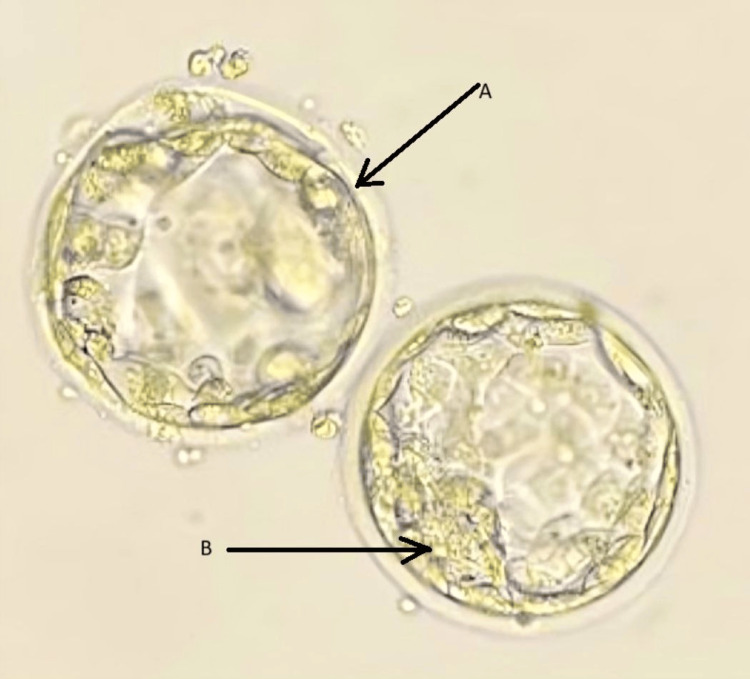
SERa-positive blastocyst of grade 3BB and 3AB selected for embryo transfer 3: size of the expansion; A: trophectoderm, B: inner cellular mass; SERa: smooth endoplasmic reticulum aggregates

Figure 2 shows day 5 frozen embryos transferred on the scheduled day of embryo transfer.

Follow-up and outcome

After embryo transfer, the female was advised to avoid any strenuous activities, take adequate rest, and all prescribed medications. Subsequently, she was advised to visit for a follow-up. The urine pregnancy test showed a positive result, and subsequently, serum β-hCG was confirmed with a value of 900 mlU/ml. A few weeks later, during a follow-up sonography, it was found that the fetus was a twin with a normal growth rate. A girl weighing 2.4 kgs and a boy weighing 2.8 kgs were delivered by C-section 35 weeks later, both without any physical or mental abnormalities.

## Discussion

According to Siddhartha et al., the amount of gonadotropin administered, the duration of ovarian stimulation, and the serum estradiol concentration on the day of ovulation trigger all positively correlate with the existence of SERa+ in MII oocytes [12]. Otsuki et al. stated that the presence of SERa in an oocyte could be indicative of prolonged cytoplasmic maturation [13]. Bianchi et al. noted that, in an ultrastructural analysis, the amount of mitochondrial smooth endoplasmic reticulum aggregates in MII oocytes that have undergone in vitro aging has significantly decreased [14]. Therefore, oocyte aging can be interpreted in at least two ways: the first is in cases of advanced maternal age and the second is in vitro aging as a result of prolonged culture during ART operations [15].

Due to possible disruptions in spindle size and cortical actin structure in SERa-positive oocytes, Dal Canto et al. found an increase in difficulties related to meiotic and mitotic division, which could ultimately result in abnormal embryo division [16]. Similarly, in our case, there was an embryonic arrest at the cleavage stage.

The Istanbul Consensus Workshop disapproved of SERa-containing the use of oocytes in 2011 because research indicated a significant risk of deformity in children produced using these oocytes. Furthermore, SERa has been linked to a noteworthy occurrence of birth abnormalities, prenatal issues, and imprinting disorders [17]. Although several studies have documented the births of healthy babies derived from SERa-positive cycles (in which there is a minimum of a single SERa-positive oocyte within a cohort) or even from a cohort comprised of all SERa-positive oocytes, the issue of discarding SERa-positive oocytes is still debatable [18-21]. Therefore, we obtained detailed informed consent from our patient prior to transferring SERa+ embryo.

According to Wang et al., the occurrence of SERa was reported in the literature. These include the extended MII introduced by an early LH surge or a symptom of prolonged cytoplasmic maturation before the LH surge is triggered during the controlled ovarian stimulation cycle. It has also been suggested that there is a correlation between high estradiol levels and the presence of SERa in the oocyte. Furthermore, patients who have had multiple unsuccessful IVF cycles have been observed to exhibit a recurring presence of SERa; our patient also had a history of two unsuccessful IVF cycles, which may have contributed to the appearance of SERa [21]. Fang et al. [22] concluded that the presence of SERa in the oocyte resulted in cleavage failure, and according to the above observation, it also affects the cleavage rate in such a way that the cleavage rate of SERa+ oocytes was much lower than the cleavage rate of normal ICSI [22].

Tevkin et al. [23] stated that the presence of GM-CSF in the culture medium allows embryos to grow and develop normally, controls how embryos interact with endometrial cells, and improves trophoblast adhesion and proliferation. The data align with the findings of several other authors and demonstrate that the inclusion of recombinant GM-CSF in the culture medium improves implantation, approaches the natural conditions of human embryo cultivation in vitro, and may have a positive impact on the effectiveness of ART techniques [23].

## Conclusions

This study concludes that embryos originating from SERa+ oocytes may exhibit normal development and positive pregnancy can occur despite a reduced cleavage rate. The fact that the female participant in this case had two unsuccessful IVF cycles in the past may have contributed to the SERa+ content of the oocyte. Thus, further investigation is required to obtain a more comprehensive understanding of the elements that contribute to the occurrence and reappearance of SERa in oocytes.
